# Morphology-Controlled Synthesis of Hematite Nanocrystals and Their Optical, Magnetic and Electrochemical Performance

**DOI:** 10.3390/nano8010041

**Published:** 2018-01-15

**Authors:** Bangquan Li, Qian Sun, Hongsheng Fan, Ming Cheng, Aixian Shan, Yimin Cui, Rongming Wang

**Affiliations:** 1 Department of Physics, Beihang University, Beijing 100191, China; bangquanli@buaa.edu.cn (B.L.); hsfan@buaa.edu.cn (H.F.); cuiym@buaa.edu.cn (Y.C.); 2 Institute of Solid State Physics, Shanxi Datong University, Datong 037009, China; 3 Beijing Institute of Space Mechanics and Electricity, Beijing 100094, China; sunqian2345216@163.com; 4 Beijing Key Laboratory for Magneto-Photoelectrical Composite and Interface Science, School of Mathematics and Physics, University of Science and Technology Beijing, Beijing 100083, China; chengming@buaa.edu.cn (M.C.); sax2005@163.com (A.S.)

**Keywords:** hematite, hollow structure, ferromagnetic behavior, supercapacitor, cycling stability

## Abstract

A series of α-Fe_2_O_3_ nanocrystals (NCs) with fascinating morphologies, such as hollow nanoolives, nanotubes, nanospindles, and nanoplates, were prepared through a simple template-free hydrothermal synthesis process. The results showed that the morphologies could be easily controlled by SO_4_^2−^ and H_2_PO_4_^−^. Physical property analysis showed that the α-Fe_2_O_3_ NCs exhibited shape- and size-dependent ferromagnetic and optical behaviors. The absorption band peak of the α-Fe_2_O_3_ NCs could be tuned from 320 to 610 nm. Furthermore, when applied as electrode material for supercapacitor, the hollow olive-structure exhibited the highest capacitance (285.9 F·g^−1^) and an excellent long-term cycling stability (93% after 3000 cycles), indicating that it could serve as a candidate electrode material for a supercapacitor.

## 1. Introduction

At nanoscale, metal oxides have been identified to exhibit different optical, magnetic and electrochemical performance from their bulk counterparts [1,2,3]. The synthesis of metal oxide nanocrystals (NCs) and their composites have attracted significant research interest for both fundamental investigation and practical applications [4,5]. As the most stable phase of iron oxides, hematite (α-Fe_2_O_3_) shows an n-type semiconductor (E_g_ = 2.1 eV) property in ambient conditions [6,7]. Due to the size and shape effect, the α-Fe_2_O_3_ NCs offer various physical and chemical properties. In virtue of their environmental friendliness, non-toxicity, excellent thermal stability, good corrosion resistance and low cost, they have been extensively investigated in the fields of catalysts [8,9], gas sensors [10], environment protection [11] and energy storage [12].

Recently, clean and renewable energy materials present promising applications in solving the problems of the energy shortage and environmental pollution. The supercapacitor is one of the most ideal and promising energy storage devices [13,14,15,16]. Hematite NC has large theoretical specific capacitance (3625 F·g^−1^), which favors it as electrode materials for supercapacitors [17]. For instance, porous nanoflowers [18], nanorods [19] and 3D spinous iron oxide materials [20] facilitate the faradic reaction toward electrolytes and deliver high specific capacitances and long cycle life. In addition, α-Fe_2_O_3_ nanomaterials exhibit excellent optical and magnetic properties, which could be adopted to meet the specific requirements of the desired applications. For example, the hollow NCs show a size-dependent blue shift, strong visible-light and broad infrared absorption phenomenon [21]. The α-Fe_2_O_3_ nanomaterials show ferromagnetic behavior and high coercivity in magnetic hysteresis measurements [22,23]. The rational design and synthesis of nanomaterials with different morphology have become an effective strategy to improve their optical, magnetic and electrochemical properties [24].

Though many methods have been developed to control the morphology, it is a great challenge to fabricate hematite NCs with the controlled size and morphology [25,26,27]. Among the different morphologies of nanomaterials, hollow structures have attracted much attention for their peculiar characteristics such as low density, high porosity and large specific surface area [28,29,30]. The reported hollow hematite spheres are fabricated by using the template method or the surfactant-assisted solvothermal method [31,32]. Though the as-prepared hollow structures exhibit confined morphology and narrow size distribution, the complex synthesis steps including the preparation and removal of templates as well as refining surfactant have blocked the scale-up preparation and practical applications. Recently, template-free and surfactant-free synthesis of nanomaterials has been adopted frequently because of its intrinsic advantages such as low cost and relatively simple steps [33].

Here, we adopted an environmentally friendly template-free hydrothermal synthesis method to prepare uniform hollow olive-shaped nanostructures as well as nanotubes, nanospindles and nanoplates on a large scale. Our results demonstrated that the morphology transformations were easily controlled by the SO_4_^2−^ and H_2_PO_4_^−^. Furthermore, we found that the optical absorption and magnetic properties of the α-Fe_2_O_3_ NCs were associated with their sizes and shapes. Besides, electrochemical properties of as-prepared hematite were investigated.

## 2. Experimental Details

### 2.1. Materials

FeCl_3_·6H_2_O, Na_2_SO_4_, and NaH_2_PO_4_ were purchased from LANYI REAGENT Ltd. (Beijing, China). All reagents were analytic grade and employed as received without further purification.

### 2.2. Methods

In a typical synthesis [34], ferric chloride (FeCl_3_·6H_2_O, 0.648 g) and Na_2_SO_4_ (0.009 g) were dissolved in 60 mL distilled water and followed by the addition of NaH_2_PO_4_ (0.009 g) under magnetic stirring to form clear precursor solution. The mixture was then transferred to a 75 mL Teflon-lined autoclave and maintained at 230 °C for 12 h. After the autoclave was cooled to room temperature, the products were obtained by centrifuging and washed with ethanol and water for several times. The powders were dried at 60 °C for 6 h for further characterization. 

The process parameters have a significant effect on the microstructures of NCs. To gain further insight into morphology evolution of NCs, the samples of nanotubes, nanospindles, nanoplates were prepared with the additional NaH_2_PO_4_ and Na_2_SO_4_ as well as the reaction time (see in Table S1).

### 2.3. Characterization and Measurement

The X-ray diffraction (XRD) patterns of the as-synthesized products were recorded on using a Bruker D8 Advance instrument (Karlsruhe, Germany) equipped with a Cu Kα radiation source, operating at 40 kV and 40 mA, and with a Ni filter. X-ray photoelectron spectroscopy (XPS) was further performed by using an ESCALAB MK II (Al Kα photon source, C1s 284.8 eV, VG Scientific LTD, London, UK). The specific surface areas of the products were determined by N_2_ adsorption–desorption isotherm measurements (Micrometrics, ASAP 2020, Concord, NH, USA). The morphology and structure were characterized by a Hitachi S-4800 scanning electron microscope (SEM, Tokyo, Japan) with a cold field emission gun and by a JEOL JEM-2100F transmission electron microscope (TEM, Tokyo, Japan) and high-resolution TEM (HRTEM) operated at 200 kV. The specimen for the SEM studies was prepared by dispersing the powder sample on the silicon substrate, while that for TEM and HRTEM investigations was obtained by dispersion of the as-synthesized products in ethanol and then a drop of the suspension was placed on a copper grid and dried in air.

UV-vis adsorption spectra were recorded using a Hitachi 3010 spectrophotometer (Tokyo, Japan). Magnetic properties of the as-synthesized powder samples were measured using a superconducting quantum interference device (SQUID) magnetometer (Quantum Design, San Diego, CA, USA). The electrochemical properties of the as-obtained electrodes were investigated by using conventional three-electrode system (CHI760e, Shanghai Chenhua, Shanghai, China). To prepare the working electrodes, the as-synthesized active materials were mixed with carbon black and polyvinylidene fluoride (PVDF) with a mass ratio of 7:2:1 in *N*-methyl-2-pyrrolidinone. The mixture was pasted on the cleaned nickel foam (area ~1 cm^2^) and dried in an oven at 60 °C for 12 h. The dried electrode was pressed using a hydraulic press at a pressure of 8 MPa. The loading mass of the active material on nickel foam is about 3 mg. The Ag/AgCl electrode and the Pt wire electrode were used as the reference electrode and the counter electrode, respectively. Cyclic voltammetry (CV) and chronopotentiometry (CP) were performed in KOH electrolyte of 2 mol·L^−1^. In the cycling stability tests, the charge-discharge current density is 1 A·g^−1^. The electrochemical impedance spectroscopy (EIS) was also recorded on the same electrochemical workstation. The frequency range is from 100 kHz to 0.01 Hz with an AC perturbation of 5 mV.

## 3. Results and Discussion

### 3.1. Morphological and Structural Analysis

**Figure 1:**
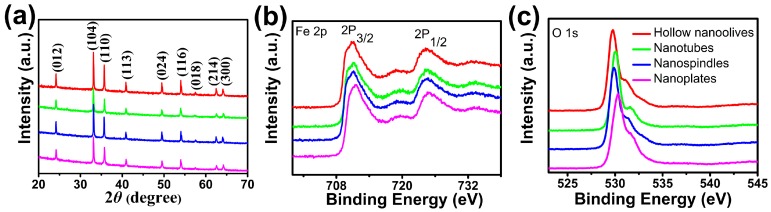
(**a**) X-ray diffraction (XRD) patterns; and X-ray photoelectron spectroscopy (XPS) spectra of (**b**) Fe 2p and (**c**) O 1s for the as-synthesized hematite (α-Fe_2_O_3_) hollow nanoolives, nanotubes, nanospindles, and nanoplates.

The crystallographic structures of the as-synthesized four products were identified to be α-Fe_2_O_3_ by X-ray diffraction (Figure 1a). The detected nine peaks are well consistent with the corundum structure α-Fe_2_O_3_ (JCPDS: 33-0664) [35], corresponding to the (012), (104), (110), (113), (024), (116), (018), (214) and (300) lattice planes of α-Fe_2_O_3_, respectively. The narrow sharp peaks suggest the high crystalline nature of the α-Fe_2_O_3_ products. No other peaks are observed, indicating the high purity of the as-prepared four samples.

XPS analyses were employed to analyze the electronic structure and surface compositions of the as-prepared samples. As shown in Figure 1b,c, two peaks centered at around 710.9 and 724.4 eV in the high-resolution spectrum of Fe 2p can be ascribed to the Fe 2p_3/2_ and Fe 2p_1/2_ spin-orbit peaks of α-Fe_2_O_3_ respectively, suggesting the Fe^3+^ ions are dominant in the product [36]. Also, the corresponding satellite peak at 718.4 eV can be solely attributed to the presence of Fe^3+^ ions of α-Fe_2_O_3_, as the binding-energy values are too high to be other oxide species of iron. In addition, the O 1s core levels show the dominant oxide peaks at around 529.0 eV, which are in good agreement with the literature values of α-Fe_2_O_3_ [37]. The Fe 2p and O 1s core levels indicate that the valence states of elements Fe and O are +3 and −2, respectively. From this result with that of XRD, we can further make sure that the products are pure α-Fe_2_O_3_.

**Figure 2:**
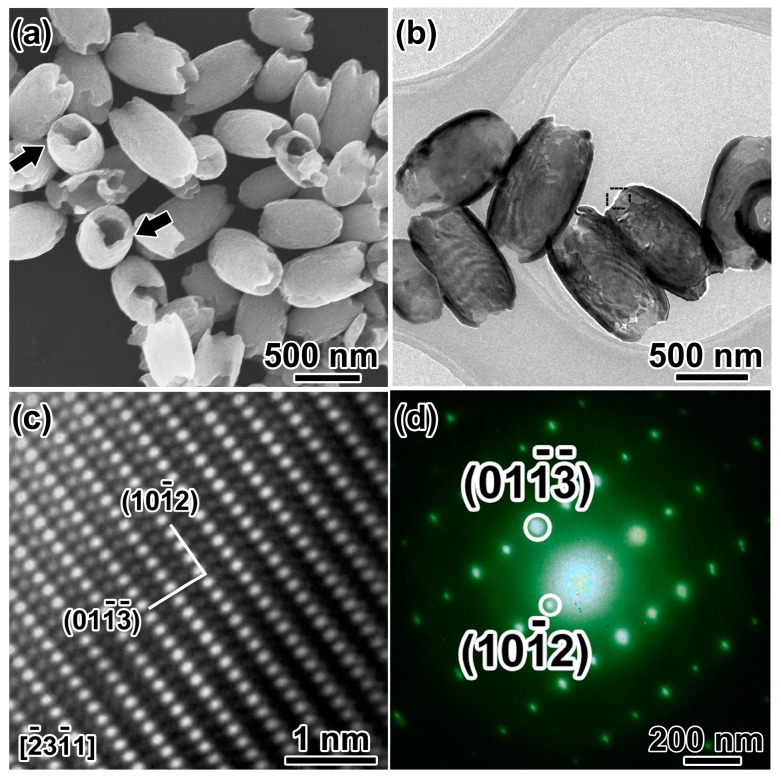
(**a**) SEM image and (**b**) TEM image of the hollow nanoolives; the black arrows show open tips; (**c**) HRTEM image and (**d**) SAED patterns of the selected area in (**b**).

Figure 2a shows an overview SEM image of the as-synthesized hematite products in the coexistence of SO_4_^2−^, and H_2_PO_4_^−^ hydrothermal synthesis. The products are olive-shaped nanostructures with open tips in both sides (~450 nm in diameter, ~800 nm in length). The NCs with the long axis perpendicular to the silicon wafer marked by the black arrow distinctly disclose the hollow structure of the α-Fe_2_O_3_ nanoolives. TEM investigations give a deeper insight into the fine microstructures as shown in Figure 2b. The strong contrast between the relatively darker edges and the brighter center of the α-Fe_2_O_3_ NCs further confirms the hollow nature. The shell thickness can be measured to be ~50 nm. Lattice resolved high-resolution transmission electron microscopy (HRTEM) in Figure 2c demonstrates the good crystallization of the sample. Lattice fringe distances were measured to be 0.367 and 0.315 nm, respectively, which can be indexed to ($10\bar{1}2$) and ($01\bar{1}\bar{3}$) planes of corundum hematite along ($\bar{2}3\bar{1}1$) zone axis. Figure 2d shows the selected area electron diffraction (SAED) pattern of the selected area in Figure 2b, which matches well with the HRTEM results and demonstrates the single-crystal structure of the α-Fe_2_O_3_ nanoolives.

Upon careful observation, the open tips were found coarse with some voids, which might imply a corrosion process happening during the formation of the hollow structures. Thus, controlled experiments have been done to study the formation of the olive-shaped hollow α-Fe_2_O_3_. Influence of the ions in the precursor had been investigated.

Tube-like structures (~150 nm in diameter and ~400 nm in length) were obtained when the concentrations of iron ions in the precursor were halved (Figure S1). The open tips of the tubes were much rougher and broken. When the ratios of H_2_PO_4_^−^ and SO_4_^2−^ to Fe^3+^ in the precursor were increased, corrosion behaviors were more obvious.

**Figure 3:**
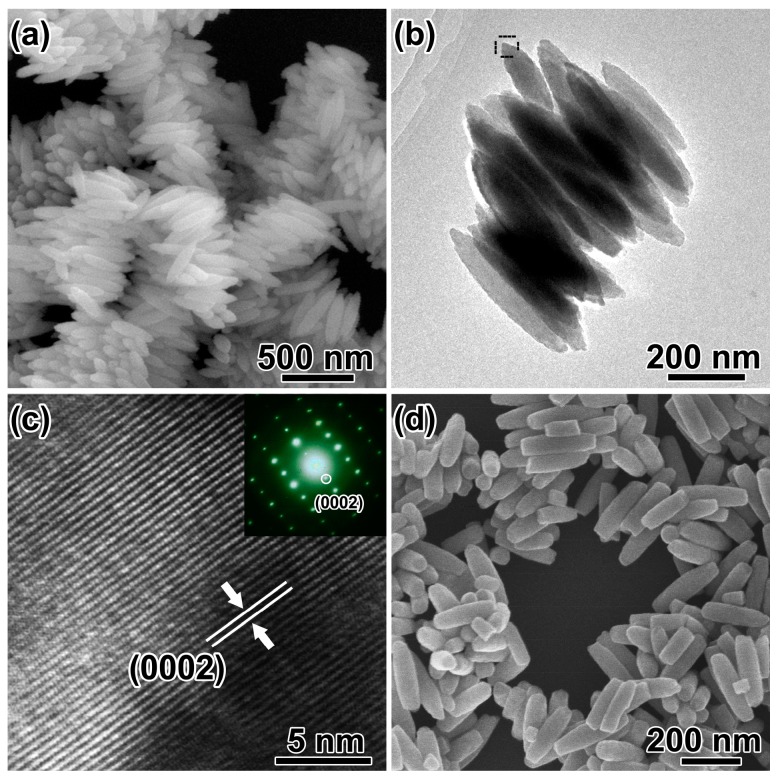
(**a**) SEM image and (**b**) TEM image of the as-synthesized nanospindles; (**c**) HRTEM image and SAED pattern (inset) of the selected area in (**b**); (**d**) SEM image of the products with solvothermal treatment of 48 h.

Without addition of SO_4_^2−^ in the reaction system, spindle-like hematite structures with diameter of ~150 nm and length of ~400 nm were obtained (Figure 3a). The spindles aggregated together by sharing their long axis and no voids were observed (Figure 3b). HRTEM image (Figure 3c) and SAED pattern (inset of Figure 3c) were taken from the spindle marked with black frame in Figure 3b. The spacing distance of the lattice fringes is measured to be 0.677 nm, corresponding to the (0002) planes of α-Fe_2_O_3_. The long axis of the spindles is along the [0001] direction of hematite, which could be the growth direction. With solvothermal treatment time increased to 48 h, the spindles with flat tips were obtained as shown in Figure 3d. The atomic coordination number is small, and the atoms at the tip would be corroded with the extension of time. Based on the discussion above and the literature [38], H_2_PO_4_^−^ are essential for the formation of spindle-like structure.

**Figure 4:**
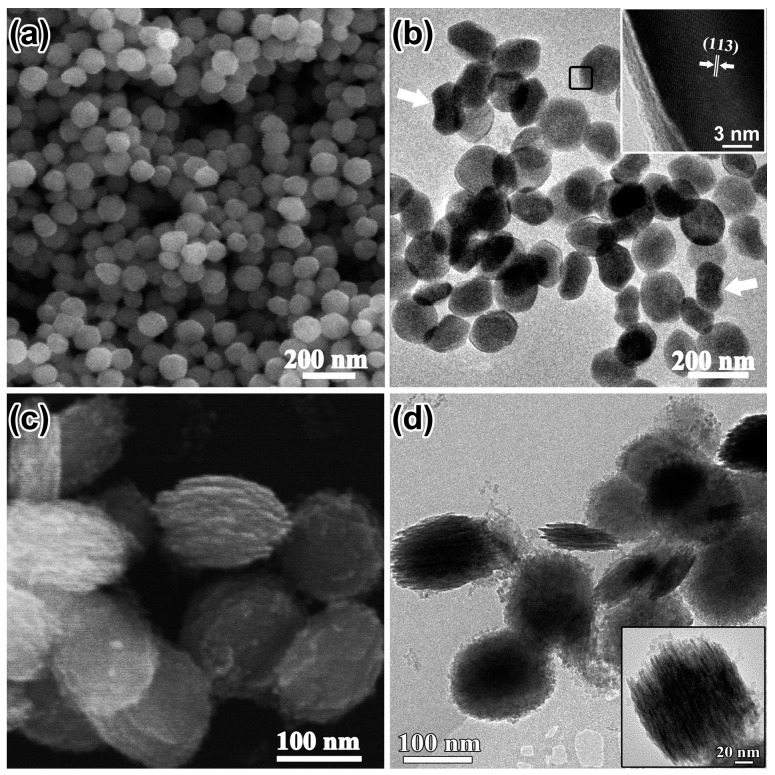
(**a**) SEM image; (**b**) TEM image and HRTEM image (inset); the white arrows in (**b**) show nanoplates with edges perpendicular to the carbon grid; (**c**) SEM image of the products synthesized by decreasing solvothermal treatment time to 3 h; (**d**) corresponding TEM image and a magnified image (inset) of a typical plate-like precursor.

The concentration of SO_4_^2−^ ions, which might promote the coordination-assisted dissolution process, was also investigated without addition of H_2_PO_4_^−^. Figure 4a shows the as-prepared products of α-Fe_2_O_3_. NCs with a clear shape edge (150 nm in diameter). TEM investigation revealed that the α-Fe_2_O_3_ NCs were plate-like (marked with black frame) and some of them with the edge perpendicular to the carbon grid (marked with white arrows) also proved the plate-like morphology of the products (Figure 4b). The nanoplates precursors with discs composed of tiny aggregated particles parallel to each other were found when the reaction time was decreased to 3 h (Figure 4c,d). The time evolution confirmed that the nanoparticles shown in Figure 4a were plate-like structures.

To conclude, the phosphate ions have been reported to act as a shape controller to induce the anisotropic growth of α-Fe_2_O_3_ [38]. However, sulfate ions favor the dissolution of α-Fe_2_O_3_ because of their coordination effect with ferric ions [39]. Phosphate and sulfate ions can be adsorbed on the surface of α-Fe_2_O_3_, which could effectively prevent the detachment of iron atoms on the surfaces. The adsorption is very weak on the (0001) plane, so NCs grow along [0001] direction, which induces the appearance of olive-shaped structure. Although the adsorption of the phosphate and sulfate ions to α-Fe_2_O_3_ crystal planes is similar, the adsorption affinity of sulfate on α-Fe_2_O_3_ is much weaker than that of phosphate. Phosphate ions play a much more crucial role than sulfate ions in the formation of olive-shaped NCs. Under acidic conditions at a high reaction temperature, the tips of olive-shaped NCs may begin to dissolve. The dissolution occurs along the [0001] direction, as the (0001) plane is almost entirely exposed to solution. Therefore, in the presence of phosphate ions, the α-Fe_2_O_3_ NCs would grow along the [0001] direction, sulfate ions could coordinate with iron ions and lead to the “corrosion” of the Fe_2_O_3_. The hollow structures are formed. With the presence of phosphate ions, and the absence of sulfate ions, only spindle solid nanoparticles are formed. In contrast, duo to sulfate ions’ coordination effect with ferric ions and corrosion effect, the small nanoparticles gather to form the nanoplates.

### 3.2. Optical Properties

**Figure 5:**
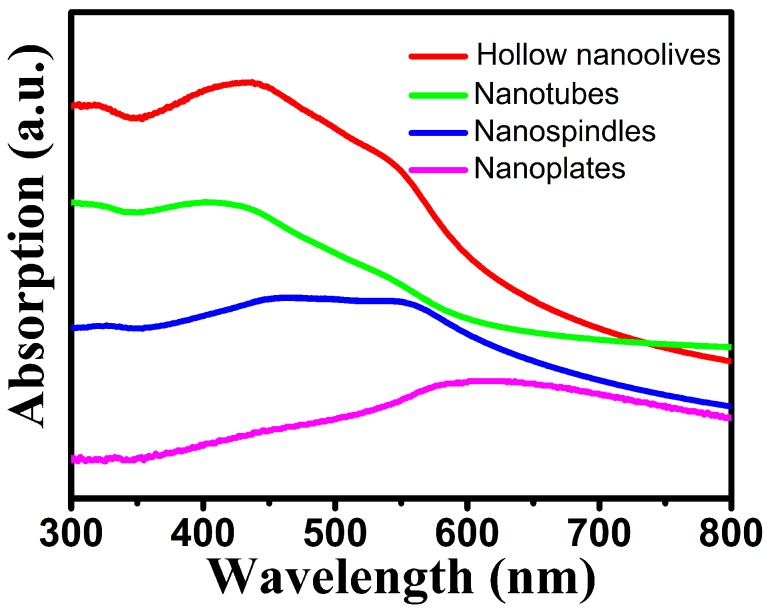
UV-vis spectra of different-shaped hematite NCs.

Hematite nanomaterials have been used widely as ultraviolet ray absorbents and photocatalyst [40]. The optical properties of metal NCs are determined by the size, shape, composition, and dielectric properties of NCs and their local environment. It is well-known that the iron oxides have three types of electronic transitions occurring in the optical absorption spectra: the ligand to metal charge-transfer transitions (LMCT), the ligand field transitions (d-d transitions) and the pair excitations [41]. As shown in Figure 5, hollow nanoolives and nanotubes have three broad bands around 320, 432 and 546 nm. The band at around 432 nm has a little blue-shifted as the length of hollow NCs reduces and the morphology transforms from hollow nanoolives to nanotubes, which can be attributed to the size of the hematite nanoparticles. For the band at 546 nm, no distinct tendency of peak shift is observed but the intensity is largely decreased, which is consistent with the reported hematite hollow NCs [42]. The nanospindles have broad band around 450 and 560 nm, while the nanoplates only have a broadband around 610 nm due to the electron transitions from valence band to conduction band which closely matches the value reported in the literature [43]. The absorption spectra of the nanospindles and nanoplates were dependent on their morphology. The wider spectral absorption may enhance its solar harvesting capability for visible light.

### 3.3. Magnetic Properties

**Figure 6:**
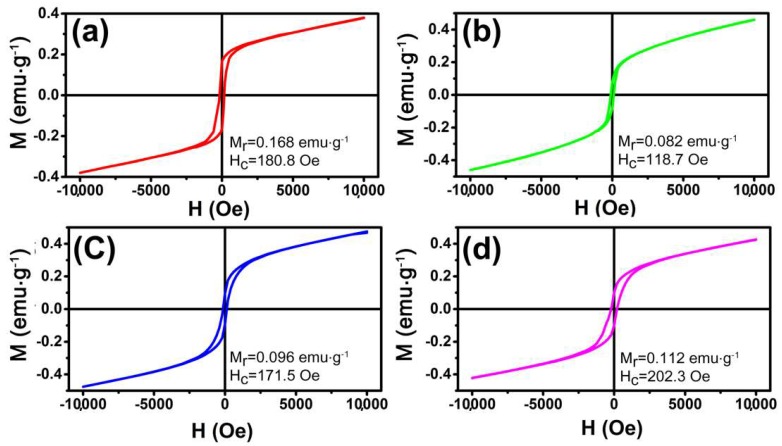
Magnetic hysteresis loops of the α-Fe_2_O_3_: (**a**) hollow nanoolives; (**b**) nanotubes; (**c**) nanospindles; (**d**) nanoplates.

The magnetization was investigated using a SQUID magnetometer. It is well known that the magnetic properties of materials were highly sensitive to the morphology, size, and crystal structure of the as-synthesized samples, which are different from those of bulk materials. Figure 6 shows the magnetic hysteresis loops of these samples at 300 K in the applied magnetic field in the range of −10 to 10 kOe. It is noted that the hysteresis loops do not reach saturation even up to the maximum applied magnetic field. The hysteresis loops indicate that the as prepared α-Fe_2_O_3_ NCs show ferromagnetic behaviors at room temperature which is similar to the reported cases in the literature [22]. The remnant magnetization (*M*_r_) of olive-shaped NCs with coercivity (*H*_c_) of 180.8 Oe is 0.168 emu·g^−1^, which is much higher than that of nanotube (0.082 emu·g^−1^). The higher *H*_c_ and *M*_r_ of the sample may be attributed to the larger particle size. In addition, the nanoplates sample displayed a remnant magnetization of 0.112 emu·g^−1^ and a coercivity of 202.3 Oe, both higher than that of nanotubes and nanospindles, as shown in Figure 6d. It must be owing to the unique morphology of tiny aggregated particles parallel to each other [44]. Therefore, the magnetization also has a shape-depended behavior [45].

### 3.4. Electrochemical Properties

**Figure 7:**
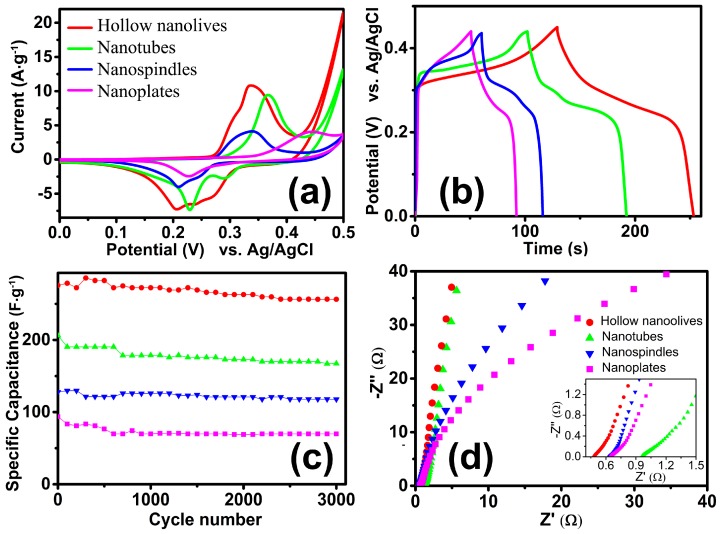
Electrochemical performance of Hollow nanoolives (red), Nanotubes (green), Nanospindles (blue) and Nanoplates (Magenta) in a three-electrode system. (**a**) CV curves; (**b**) Galvanostatic charge–discharge curves; (**c**) Long-term cyclic performances at 1 A·g^−1^; (**d**) Nyquist plots.

The electrochemical properties of the as-obtained electrodes were investigated by using conventional three-electrode system. Figure 7a exhibits CV curves of the as-grown α-Fe_2_O_3_ NCs in the potential window of 0–0.50 V at a scan rate of 10 mV·s^−1^. The obvious anodic and cathodic peaks indicate the pseudocapacitance properties of the four electrodes, which demonstrate that the capacitance characteristics are mainly governed by Faradaic reactions. The reversible redox reaction is related to the conversion between Fe^3+^ and Fe^2+^, reaction is presented as follow [17]: Fe_2_O_3_ + 2e^−^ + 3H_2_O ↔ 2Fe(OH)_2_ + 2OH^−^. In the process of charge-discharge cycles, the redox current will be higher, and the ability of charge and discharge will be increased [46]. The specific capacitance is in proportion to the area under the CV curves. Figure 7a shows that the area under the CV curve for hollow olive-shaped α-Fe_2_O_3_ is apparently much larger than those of nanotubes, nanospindles and nanoplates, which indicate that olive-shaped α-Fe_2_O_3_ have a higher specific capacitance than other samples. Besides, the charge-discharge properties of the prepared electrode are also tested under current densities of 1 A·g^−1^, as shown in Figure 7b. The nonlinearity in the discharge curves shows the pseudocapacitive behavior of the electrodes. This further confirms the pseudocapacitance feature of the as-prepared α-Fe_2_O_3_ products. It is found that the as obtained α-Fe_2_O_3_ products possess excellent capacitance performance. The specific capacitance of the electrode can be calculated from the discharge curve according to *C* = (*I*∆*t)*/(*m*∆*V)*, where *C* (F·g^−1^) is the specific capacitance, *I* (mA) is the discharge current, Δ*t* (s) is the discharge time, *m* (g) is the mass of the active material in the electrode and Δ*V* (V) is the potential change during the discharge. Remarkably, the specific capacitance of the hollow olive-shaped NCs (275.7 F·g^−1^) are much higher than that of the nanotubes (206.3 F·g^−1^), nanospindles (127.8 F·g^−1^) and nanoplates (93.7 F·g^−1^). To further verify the electrochemical properties of hollow nanoolives, Figure S2a present the typical CV curves of hollow olive-shaped NCs at different scan rates. With the increase of the scan rate, anodic and cathodic peaks shift continuously to higher and lower potentials, respectively, because of the limited ion diffusion rate in the electrolyte [47]. The charge-discharge curves at different current density are shown in Figure S2b. The near symmetric charge and discharge curves reveal excellent electrochemical reversibility properties. The corresponding specific capacitance was 275.7, 269.8, 257.9, 229.8 and 218.3 F·g^−1^ at 1, 1.5, 2, 4 and 6 A·g^−1^ current density, respectively.

Figure 7c reveals the cycling performance of the as-prepared α-Fe_2_O_3_ products. In the cycling stability tests, the hollow olive-structure delivers the highest specific capacitance (285.9 F·g^−1^) and an excellent long-term cycling stability (93% after 3000 repetitive cycles), which is superior to many previously reported systems, such as ribbon shaped [48], flowerlike [18], red blood cell shaped [49] hematite, even to FeS_2_@Fe_2_O_3_ [50] and Fe_2_O_3_ NDs@NG [51]. This is related to its large specific surface area (see in Table S1). The specific capacitance of the nanotubes, nanospindles and nanoplates maintain about 85%, 90% and 74% under the same test conditions, respectively. Thus, although the nanospindles are low in capacitance, they have good cycle stability. In order to further study the electrochemical performance of the structures, an electrochemical impedance spectroscopy (EIS) [52] was also recorded on the same electrochemical workstation. Typical Nyquist plots of the samples are presented in Figure 7d. The EIS spectrum of the electrodes was usually composed of a semicircle at the high-frequency region and a straight line at the low-frequency region. In a low frequency, the slope of the line corresponds to the Warburg impedance, which means the electrolyte diffusion occurs within the α-Fe_2_O_3_ electrodes. The nearly straight vertical line along the imaginary axis indicates a low diffusion resistance and shows good capacitor behavior, which indicates that the hollow α-Fe_2_O_3_ electrodes possess much lower diffusion resistance. The intercept at the real axis is the solution resistance of the electrochemical system, which includes the inherent resistance of the electroactive material and contact resistance at the interface between the electrolyte and the electrode. The charge transfer resistance that is also called the Faraday resistance can be evaluated from the diameter of the semicircle in the high frequency range. The minimum value of the intercept and no semicircle was observed in the electrode of the hollow olive-shaped NCs, which indicates that hollow olive-shaped NCs provides high electronic conductivity compared with other electrodes. Therefore, the hollow olive-shaped NCs exhibit good electrochemical properties for supercapacitor applications.

## 4. Conclusions

We adopted a facile template-free hydrothermal synthesis route to prepare hematite structures with different morphologies, including nanoolives, nanotubes, nanospindles, and nanoplates. The particle size and shape have a remarkable effect on the optical and magnetic properties. The as-prepared NCs exhibited different absorption bands in the ultraviolet and visible regions. In addition, they also show different ferromagnetic behaviors at room temperature. When used as a supercapacitor electrode, the hollow olive-shaped exhibited high capacity (285.9 F·g^−1^), which can be attributed to the large number of active sites provided by unique spatial structure. The cycling test revealed that this material possesses excellent cycling stability during 3000 cycles. These results indicate that the hollow nanoolive samples can be a promising candidate material in energy storage fields.

## Supplementary Materials

The following are available online at www.mdpi.com/2079-4991/8/1/41/s1, Figure S1: SEM images of the nanotubes, Figure S2: (a) CV curves of the hollow olive-shaped α-Fe_2_O_3_ electrodes at different scan rates; (b) Galvanostatic charge-discharge curves of the hollow olive-shaped α-Fe_2_O_3_ electrodes at various current densities, Table S1: The morphologies and BET surface areas of α-Fe_2_O_3_ under different reaction conditions.
