# Supplementary material for: Morphology-Controlled Synthesis of Hematite Nanocrystals and Their Optical, Magnetic and Electrochemical Performance
Source: Nanomaterials (Basel). 2018 Jan 15;8(1):41. doi: 10.3390/nano8010041 (PMC5791128; doi:10.3390/nano8010041)
Supplement: Supplementary file 1 [file nanomaterials-08-00041-s001.pdf]

nanomaterials

Supplementary Information

Morphology-Controlled Synthesis of Hematite Nanocrystals and Their Optical, Magnetic and Electrochemical Performance

Bangquan Li [1,2], Qian Sun [3], Hongsheng Fan [1,4], Ming Cheng [4], Aixian Shan [4], Yimin Cui [1] and Rongming Wang [4,*]

[1] Department of Physics, Beihang University, Beijing 100191, China; bangquanli@buaa.edu.cn (B.L.); hsfan@buaa.edu.cn (H.F.); cuiym@buaa.edu.cn (Y.C.)
[2] Institute of Solid State Physics, Shanxi Datong University, Datong 037009, China
[3] Beijing Institute of Space Mechanics and Electricity, Beijing 100094, China; sunqian2345216@163.com
[4] Beijing Key Laboratory for Magneto-Photoelectrical Composite and Interface Science, School of Mathematics and Physics, University of Science and Technology Beijing, Beijing 100083, China; chengming@buaa.edu.cn (M.C.); sax2005@163.com (A.S.)
* Correspondence: rmwang@ustb.edu.cn; Tel.: +86-133-9157-1538

Received: 23 December 2017; Accepted: 11 January 2018; Published: Date

Table S1. The morphologies and BET surface areas of α-Fe_2O_3 under different reaction conditions.

Morphology	BET (m^2/g)	$FeCl_3·6H_2O$(g)	NaH_2PO_4 (g)	Na_2SO_4 (g)	Temperature (°C)
Hollow nanoolives	33.67	0.648	0.009	0.009	230
Nanotubes	30.08	0.324	0.009	0.009	230
Nanospindles	23.57	0.324	0.009	0	230
Nanoplates	14.19	0.324	0	0.009	230

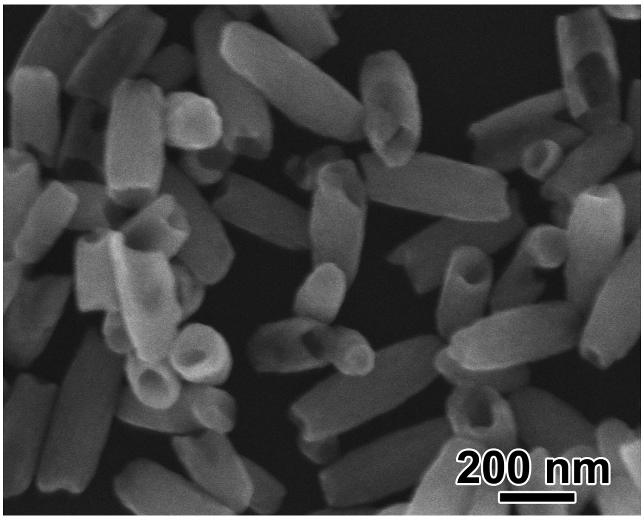

Figure S1. Scanning electron microscope (SEM) image of the nanotubes.

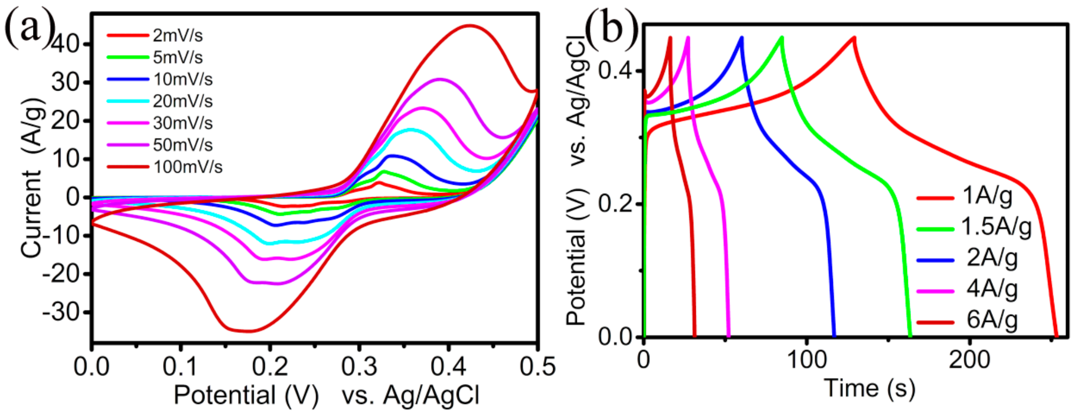

Figure S2. (**a**) Cyclic voltammetry (CV) curves of the hollow olive-shaped α-Fe_2O_3 electrodes at different scan rates; (**b**) Galvanostatic charge-discharge curves of the hollow olive-shaped α-Fe_2O_3 electrodes at various current densities.

© 2018 by the authors. Submitted for possible open access publication under the terms and conditions of the Creative Commons Attribution (CC BY) license (http://creativecommons.org/licenses/by/4.0/).
